# Thymic Tumours in Children

**DOI:** 10.3390/pediatric14010001

**Published:** 2021-12-23

**Authors:** Aleksandra Napieralska, Leszek Miszczyk

**Affiliations:** Radiotherapy Department, MSC National Research Institute of Oncology Gliwice Branch, 44-102 Gliwice, Poland; Leszek.miszczyk@io.gliwice.pl

**Keywords:** thymoma, thymic carcinoma, surgery, radiotherapy, chemotherapy, children, rare diseases

## Abstract

Thymomas are very rare neoplasms in children and they represent less than 1% of mediastinal tumours in the paediatric population. The aim of our study was to assess the long-term treatment results of children with thymic tumours. A total number of eight children (four boys and four girls) with thymic tumours were identified. Median age at diagnosis was 7 years. In seven of them, thymoma was diagnosed; in one, a thymic carcinoma was diagnosed. In five of them, the WHO type was assessed: in two of them, the B1 type was found; in one, B2 was found; in one, AB was found, and in one, C was found. In all but one, surgery was the first-line treatment, but six patients had only partial resection. One patient started treatment with chemotherapy and four others received chemotherapy after the surgery. Radiotherapy was applied in six patients, with a median total dose of 37.5 Gy. Follow-up ranged from 8.5 to 273.5 months, with a median of 6.1 years. During this time, four patients died: one due to progression of the disease, and in the other three, the reason for death was unknown. In all evaluated patients, complete regression was observed (100% local control). Two-, 5- and 10-year OS and PFS were 85% and 72%, 51% and 54%, 51% and 54%, respectively. Combined treatment could provide satisfactory results in thymoma patients. There is a need for further, larger studies, which could help to establish optimal management strategies.

## 1. Introduction

Thymomas are very rare neoplasms in children and they represent less than 1% of mediastinal tumours in the paediatric population [1]. The majority of the literature presents single case studies, with only a few national or international reports and one SEER analysis performed in 2013 [2,3,4,5,6,7,8,9]. As in adults, thymic tumours in children may be asymptomatic or present with compressive or respiratory symptoms. The classification (WHO) and staging system (Masaoka–Koga) are the same as in adult tumours, but there are only a few studies on the treatment of thymic neoplasms in the paediatric population [4,6,7,8,9,10,11,12] (Table 1 and Table 2). Treatment for these tumours often requires a combination of modalities, including chemotherapy (CTH), radiotherapy (RTH) and surgery, based on the tumour histology and extent of disease [4]. Due to the rarity of thymic tumours in children, it is impossible to conduct prospective clinical trials. For this reason, retrospective studies such as this represent the only way to collect experience.

The aim of our study was to assess the long-term treatment results of paediatric patients with thymic tumours in a single institution during a period of 35 years.

## 2. Materials and Methods

A retrospective study of patients with thymic tumours treated in our institution between 1985 and 2019 was performed. All consecutive patients with thymic tumours and younger than 18 years old were included in the study.

The following parameters were included in the analysis: date of diagnosis, primary tumour characteristics, given curative treatment, performance status, systemic therapy, RTH characteristics—total dose, irradiated volume, early and late toxicity, local response, date and reason of death. Missing dates of deaths were obtained from the Polish National Cancer Registry. The study was approved by the ethical committee of the MSC National Research Institute of Oncology in Gliwice (number: KB/430-166/21). 

The criteria for diagnosing thymoma or thymic carcinoma used were as established by the WHO classification [10] (Table 1). Tumours were staged according to the commonly used Masaoka–Koga staging system [11,12] (Table 2). The Eastern Cooperative Oncology Group (ECOG) scale was used to classify patients’ performance status. EORTC/RTOG toxicity criteria were used to assess treatment morbidity [13]. Tumour response was classified as follows: complete regression (CR) was defined as complete disappearance of all clinical and radiological evidence of the disease, partial regression (PR) as a decrease in tumour size in clinical or radiologic evaluation and progression as an increase in lesion size or the occurrence of new lesions.

Median follow-up was estimated by Kaplan–Meier analysis with the reversed meaning of the status indicator. Overall survival (OS) was calculated from the date of disease diagnosis to the date of last follow-up or death. Progression free survival (PFS) was calculated from the date of diagnosis to the date of local/distant progression or death. The Kaplan–Meier method was used to estimate survival. Statistical analyses were performed using Statistica 12.0.

## 3. Results

A total number of eight children (four boys and four girls) with thymic tumours were identified. Median age at diagnosis was 7 years (range 1 to 18). In all the cases, the diagnosis was based on diagnostic imaging and pathologic examination of the tumour tissue samples obtained during the surgery. In four cases, the tumour was found on computed tomography (CT) of the chest, and in four cases (treated in the earlier years of a study), it was found on chest X-ray. Mean tumour dimensions were 82 × 62 × 93 mm and the tumours ranged from 50 to 135 mm in the greatest dimension. In three patients, the tumour showed contrast enhancement; in four cases, the tumour was constricting nearby organs, and in one case, infiltration of surrounding tissues was described. In one patient, lung metastases were diagnosed simultaneously with the primary tumour.

All patients had histopathologic confirmation of thymic neoplasm: in seven of them, thymoma was diagnosed; in one, thymic carcinoma was diagnosed. In five of them, the WHO type was assessed: in two of them, the B1 type was found; in one, B2 was found; in one, AB was found, and in one, C was found. Tumours were staged according to the commonly used Masaoka–Koga staging system in five cases: stage II in one, stage III in three and stage IV in one.

The ECOG scale was used to classify patients’ performance status and all but one patient showed a good performance status at the time of the diagnosis (ECOG 0-1). Symptoms of the disease were present in five of them and lasted for 1 to 24 months: weakness in three of them, dyspnoea in one, cough in two, weight loss in two and fever in two. Other symptoms presented individually were: doubled vision, oedema of the neck and face and palpable neck tumour. In one patient, diagnostic was introduced due to a lung infection. None of them presented with myasthenia.

All the patients were treated with radical intention. The characteristics of the treatment used in particular patients are presented in Table 3. In all but one, surgery was the first-line treatment; however, only in two cases was the complete removal of the tumour performed, while other patients had partial resection. One patient started treatment with CTH and four others received CTH after the surgery. In one patient, CTH was combined with RTH. Systemic treatment applied was: ADOC (doxorubicin 40 mg/m^2^ + cisplatin 50 mg/m^2^ + vincristine 0.6 mg/m^2^ + cyclophosfamid 700 mg/m^2^) in three patients; cisplatin + adriamicin + bleomycin + encorton in two patients, VIP (etoposide 75 mg/m^2^ + cisplatin 20 mg/m^2^ + ifosfamide 1.2 g/m^2^) in one patient and PACE (cisplatin 50 mg/m^2^ + doxorubicin 50 mg/m^2^ + cyclophosfamid 500 mg/m^2^) in one patient. In two of them, ADOC was followed by VIP, and in one, it was followed by cisplatin + adriamicin + bleomycin + encorton. Usually, six cycles of CTH were applied.

RTH was applied for a median total dose of 37.5 Gy. Radiotherapy characteristics are presented in Table 4. In all patients, the conformal technique (two or three fields technique) was used. The irradiated volume (CTV, clinical target volume) included mediastinum in four patients, combined with a boost on lung metastasis in a stage IV patient and with a tumour bed boost and chest lymph node irradiation in one patient, tumour in one patient and tumour bed in one patient. Planned target volumes (PTVs) were created by adding additional margins to CTV in order to correct for inaccuracies in the delivery system (set-up margins) or interfraction and intrafraction of organ motions.

Follow-up ranged from 8.5 to 273.5 months, with a median of 6.1 years. During this time, four patients died: one due to progression of the disease, one due to complications of autologous stem cell transplantation during the treatment of lymphoma, and in two others (treated in 1985 and in 1991), the reason for death was unknown but, during the last visit, no evidence of thymoma was present. During this time, the epidemiology department did not collect data regarding the reason for death and, due to ethical reasons, we did not decide to contact the families of the patients. All others are still alive and free of the disease. After treatment, the size of the tumour was assessed based on a comparative radiologic analysis—images taken before and after the treatment were compared for all but one case. In all evaluated patients, complete regression was observed and no local progression was found in any of the patients (100% local control). The patient with progression of the disease (lung metastases) received RTH as a salvage treatment and lived for 21 more months. Two patients had mild acute radiation reactions—one leucopoenia and one pneumonia. All patients received the planned dose and no treatment interruptions were noted. After the treatment, CT imaging revealed radiological fibrosis of the irradiated lung volume in one patient, albeit without clinical symptoms. During the follow-up, one patient (one year old at the time of thymoma diagnosis) was diagnosed with lymphoma 22 years after the treatment, which eventually was the reason for death.

**Figure 1:**
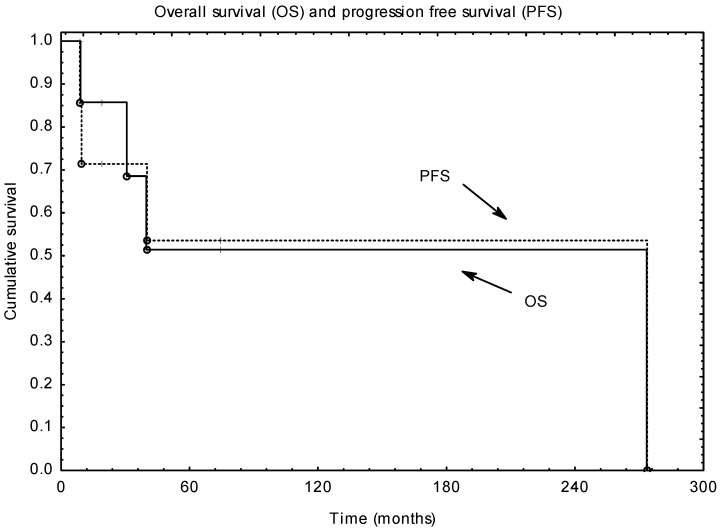
Overall and progression-free survival.

Two-, 5- and 10-year OS and PFS were 85% and 72%, 51% and 54%, 51% and 54%, respectively (Figure 1). None of analysed factors had a statistically significant impact on OS or PFS.

## 4. Discussion

Thymic neoplasms are very rare tumours in paediatric patients. The present literature concerning thymic tumours mostly comprises single case reports, with only a few larger series [2,3,4,5,6,7,8,9]. Diagnosis, as in adults, is made after biopsy or tumour resection, but could be difficult due to the rarity of this tumour in the paediatric population. Differential diagnosis of mediastinal mass usually reveals lymphoma or neurogenic tumours [4]. Patients present with nonspecific symptoms of airway obstruction, which was also observed in our study, although not all of the patients had disease signs [1,2,3,4,5,6,7,8,9]. None of the children presented with myasthenia gravis, but the coexistence of it with thymoma in children is less frequent, with an incidence of 7 to 15% [2,3,14,15]. The WHO histopathological classification and Masaoka–Koga staging systems are the basis of treatment choice in adults as well as in children [10,11,12].

Fonseca AL. et al., in their report, described the results of thymic tumours in children and found that this tumour type presents more often in males, but our group showed no sex predominance [15]. The largest study of Stachowicz-Stencel of 36 thymoma and thymic carcinoma patients showed a male predominance, with 21 male vs. 15 female patients. Moreover, Rod J., in his literature analysis, found a male predominance in the paediatric thymoma population [2,3]. The median age of patients in our study was younger than that reported in the literature (median of 12 years old) [2,3].

Surgery, possibly with R0 resection, remains the gold standard for treating all thymic neoplasms. CTH and RTH are used in patients with large or inoperable tumours, in the presence of metastases or after non-radical resections [2,3,4,5,6,7,8,9] (Table 5). Thymic carcinoma carries a poorer prognosis and chemoradiotherapy is often offered after resection [2,3,6]. RT doses used in other studies usually are within the range of 40 to 54 Gy, which corresponds to the total doses used in the majority of our patients [1,2,3,4,5,6,7,8,9]. The very young age could be the reason for implementing doses below 40 Gy in our group. A report from the European Cooperative Study Group for Pediatric Rare Tumors (EXPeRT) written by Stachowicz-Stencel T. et al. described the results of 16 patients with thymoma and 20 with thymic carcinoma [2]. Only one child with thymoma received RTH, and, at the end of the median follow-up of 5 years, only two patients (12.5%) died due to the disease. Cisplatin was the most commonly used systemic agent, but only four patients received CTH. Importantly, radical surgery (R0) was achieved in 11 of them and was the only treatment in those patients. Thymic carcinoma patients in the majority had only biopsy (80%) and received CTH (70%) and RTH (60%), with a total dose of 38 to 54 Gy, which could explain the poor result of the 5-year OS of only 21% [2]. The same observation could be made based on Ramon y Cajal S et al.’s study, with a median survival of patients with thymic carcinoma of only 8 months [8]. Moreover, Rod et al., in a report from the French Society of Pediatric Oncology, observed poor results in patients with thymic carcinoma [3]. The patient with thymic carcinoma included in our analysis had radical surgery and received postoperative RTH to 44 Gy, which resulted in a 74-month disease-free period during follow-up.

The limitations of our study are as in all studies on rare tumours—a retrospective study covering a long time period with different treatment modalities. However, due to the rarity of this tumour in the paediatric population, studies such as ours are the only way to collect the experience on this topic. Patients who lack the thymoma subtype evaluation were treated in the early years of the study (1985, 1991 and 1992), and, according to Polish medical law, tissues are stored in the pathology department for a period of 20 years and then discarded. Since the study period covered a much longer time period, these specimens were not available. We are aware that this is one of the major drawbacks of the study, but we were not able to collect the tissue samples and evaluate them for the purpose of this study due to the mentioned legal and logistical factors.

## 5. Conclusions

Combined treatment, including surgery, chemotherapy and radiotherapy, could provide satisfactory results in thymoma patients. There is a need for further, larger, multi-national or institutional analyses with long-term follow-up, which could help to provide more information for optimal management strategies and outcomes.

## Figures and Tables

**Table 1:** WHO Classification of Thymic Epithelial Tumours [10].

Type	Histopathology
A	Tumour composed of a population of neoplastic thymic epithelial cells with spindle or oval shape with no atypia and few or no non-neoplastic lymphocytes.
AB	Tumour with foci having the features of type A thymoma mixed with foci rich in lymphocytes.
B1	Tumour that resembles normal thymus, combining large expanses having an appearance almost like normal thymic cortex.
B2	Scattered plump cells with vesicular nuclei and distinct nucleoli among population of lymphocytes.
B3	Epithelial cells having a round or polygonal shape and exhibiting no or mild atypia with a minor component of lymphocytes resulting in a sheet like growth of the neoplastic epithelial cells
Thymic carcinoma (type C)	Tumour exhibiting clear-cut cytologic atypia and a set of cytoarchitectural features no longer specific to the thymus, analogous to those seen in carcinomas, lack immature lymphocytes

**Table 2:** Masaoka–Koga staging system for thymoma [11,12].

Stage	Description
I	Grossly and microscopically completely encapsulated tumour.
IIa	Microscopically transcapsular invasion.
IIb	Macroscopic invasion into thymic or surrounding fatty tissue, or grossly adherent to but not breaking through mediastinal pleura or pericardium.
III	Macroscopic invasion into neighbouring organs (i.e., pericardium, great vessel, or lung).
IVa	Pleural or pericardial metastases.
IVb	Lymphogenous or hematogenous metastasis.

**Table 3:** Characteristics of the treatment in particular patients.

Patient, Age	HP Subtype	Surgery	CTH	RTH	Results	Recurrence	FU Duration (Months), Last Status
1, 1 y/o	thymoma	Yes, N/D	No	No	CR	No	274, DND ^1^
2, 3 y/o	thymoma	Yes, NR	CRTH	Yes	CR	No	19, AND
3, 4 y/o	thymoma B1, III Masaoka	Yes, NR	Yes	Yes	CR	No	98, AND
4, 7 y/o	thymoma B2, III Masaoka	Yes, NR	Yes	Yes	CR	No	9, DND
5, 7 y/o	thymoma B1	Yes, NR	Yes	No	N/D	No	9, AND
6, 11 y/o	thymoma, II Masaoka	Yes, R	Yes	Yes	CR	No	40, DND
7, 16 y/o	thymic carcinoma, C	Yes, R	No	Yes	CR	No	74, AND
8, 18 y/o	thymoma AB, IV Masaoka	Yes, NR	No	Yes	CR	No, Metastases	10, DWD

AND—alive no disease, CR—complete regression, CTH—chemotherapy, CRTH—chemoradiation, DND—dead no disease, DWD—dead with disease, N/D—not described, NR—non-radical, R—radical, RTH—radiotherapy, TD—total dose, y/o—years old. ^1^ died due to complications of autologous stem cell transplantation during the treatment of lymphoma.

**Table 4:** Radiotherapy characteristics.

Patient	RTH	RT Indication	Energy	Irradiated Volume	Dose 1 Phase/ 2 Phase (Gy)	Total Dose (Gy)	Fraction Dose (Gy)
1, thymoma	No	-	-	-	-	-	-
2, thymoma	Yes	non-radical surgery	60Co photons	mediastinum	28.8 + 1.2	30.0	1.6, 1.2
3, thymoma	Yes	non-radical surgery	6–20 MV X photons	tumour	15.0 + 10.5	25.5	1.5
4, thymoma	Yes	non-radical surgery	6–20 MV X photons	mediastinum + tumour bed boost + chest lymph nodes	30 +10.5	40.5	1.5
5, thymoma	No	-	-	-	-	-	-
6, thymoma	Yes	transcapsular invasion	9 MV X photons	mediastinum	34.5	34.5	1.5
7, thymic carcinoma	Yes	transcapsular invasion	60Co photons	tumour bed	44.0	44.0	2.0
8, thymoma	Yes	lung metastases	9 MV X photons	mediastinum, lung metastasis	20.4 + 39.6	60.0	1.2, 1.8

**Table 5:** Thymoma and thymic carcinoma studies.

Study (Reference) Year	Number of Patients	Treatment	5-Year OS	Mean Follow-Up (Months)
Allan BJ et al. [4] 2013	23 thymoma	Not described for thymoma patients (SEER analysis)	54%	89
Stachowicz-Stencel T et al. [2] 2015	36 (16 thymoma, 20 thymic carcinoma)	100% surgery (33% radical),	Thymoma—not reported, thymic carcinoma—21%	60
Stachowicz-Stencel T et al. [6] 2010	9 thymic carcinoma	61% CTH (78% neoadjuvant),	7 patients died, 2 alive	-
Rod J et al. [3] 2014	9 (6 thymoma, 3 thymic carcinoma)	22% RTH (1 thymoma and 12 thymic carcinoma)	2 patients with thymic carcinoma died, all others alive	48
Carretto E et al. [5] 2011	9 (4 thymoma, 5 thymic carcinoma)	5 surgery (3 radical), 3 biopsy only, 6 CTH, 4 RTH	1 thymoma and all patients with thymic carcinoma died, all others alive	-
Yalçin B et al. [7] 2012	11 thymoma (6 benign, 5 invasive)	100% surgery (4 radical)	Benign and all free of disease; invasive 2 died, 3 alive	Benign—211
Ramon y Cajal S et al. [8] 1991	10 (7 thymoma, 3 thymic carcinoma)	5 CTH, 4 RTH	Thymic carcinoma, median survival 8 months	Invasive—209 (alive)
Pescarmona E et al. [9] 1992	5 thymoma	5 surgery (3 radical), 4 biopsy only 5 CTH (thymic carcinoma), 4 RTH (thymic carcinoma)	All alive, disease-free 3 months to 9 years after surgery	-
Present study—Napieralska A. 2021	8 (7 thymoma, 1 thymic carcinoma)	9 surgery, 4 CTH (invasive only), 4 RTH (invasive only)	51%	-

CTH—chemotherapy, RTH—radiotherapy.

## Data Availability

The data presented in this study are available in this article.
